# Author Correction: Electric vehicle battery chemistry affects supply chain disruption vulnerabilities

**DOI:** 10.1038/s41467-025-61370-4

**Published:** 2025-06-30

**Authors:** Anthony L. Cheng, Erica R. H. Fuchs, Valerie J. Karplus, Jeremy J. Michalek

**Affiliations:** 1 Department of Engineering and Public Policy, Carnegie Mellon University, Pittsburgh, PA USA; 2 Wilton E. Scott Institute for Energy Innovation, Carnegie Mellon University, Pittsburgh, PA USA; 3 Department of Mechanical Engineering, Carnegie Mellon University, Pittsburgh, PA USA; 4 Department of Civil and Environmental Engineering, Carnegie Mellon University, Pittsburgh, PA USA

**Keywords:** Batteries, Energy modelling, Energy security, Energy supply and demand, Energy policy

Correction to: *Nature Communications* 10.1038/s41467-024-46418-1, published online 08 March 2024

In the version of the article initially published, in Fig. 4e, the labels “Li Refining: +23% CHN” and “Total: 80% CHN” should have read “Li Refining: +22% CHN” and “Total: 78% CHN”, respectively. Additionally, the text “Sums may not add due to rounding” has been added to the figure caption. These corrections have been made to the HTML and PDF versions of the article.

