# Supplementary material for: Electric vehicle battery chemistry affects supply chain disruption vulnerabilities
Source: Nat Commun. 2024 Mar 8;15:2143. doi: 10.1038/s41467-024-46418-1 (PMC10923860; doi:10.1038/s41467-024-46418-1)
Supplement: Supplementary file 1 — Supplementary Information [file 41467_2024_46418_MOESM1_ESM.pdf]

Electric vehicle battery chemistry affects supply chain disruption vulnerabilities: Supplementary Information

Supplementary Text S1. Further Context for Choices in Electric Vehicle Battery Chemistry

Supplementary Text S1-1. Battery Materials Supply Chain Structure (Theoretical and Simplified), and Current Electric Vehicle Manufacturer Decision Making in these Supply Chains

This supplementary section contains two figures that describe the theoretical and modeled supply chains for electric vehicle battery materials, respectively. The primary difference is the removal of stockpiling and manufacturing losses, which are typically not explicitly considered in material flow analysis studies.[1–5] Instead, we note that any stockpiling or manufacturing losses are incorporated into our uncertainty bounds when we split our refining and manufacturing into battery-related and non-battery-related refining and manufacturing, respectively. This split is identified based on our trade code aggregations as described in Supplementary Text S3-3. While there may be some lag in production processes, such as lithium brines requiring up to a year to process via evaporation,[6] USGS production data is reported in terms of final production quantities (e.g. after lithium concentration), as described in Supplementary Text S3-2. Using this model, we then map current electric vehicle manufacturer decision making onto the modeled supply chain.

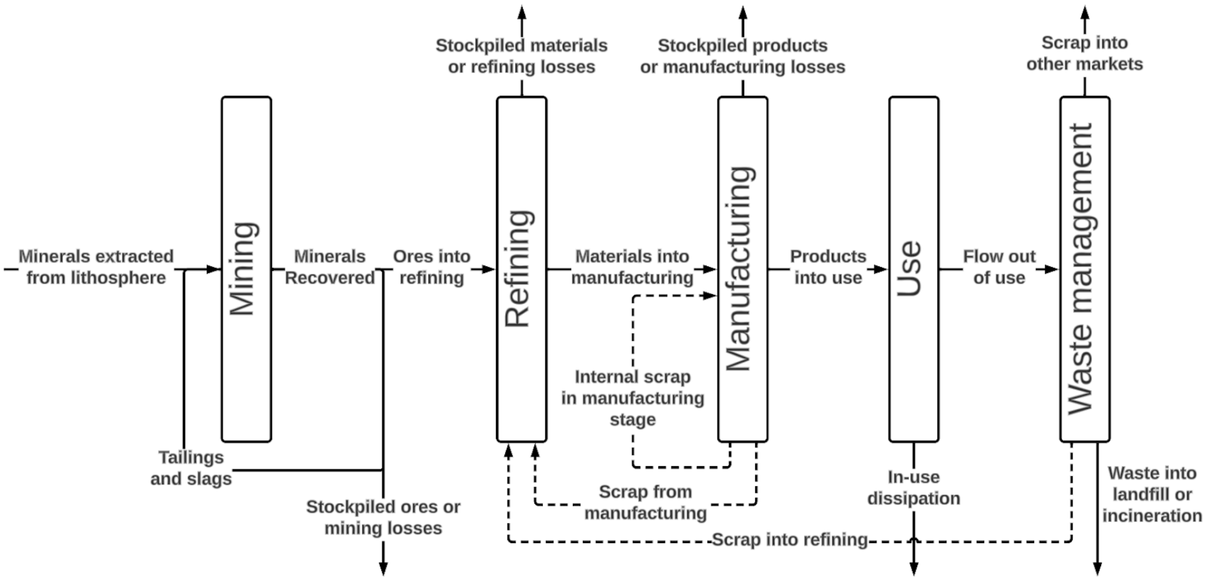

Supplementary Fig. 1. The theoretical complete supply chain flows for a battery material.[2]

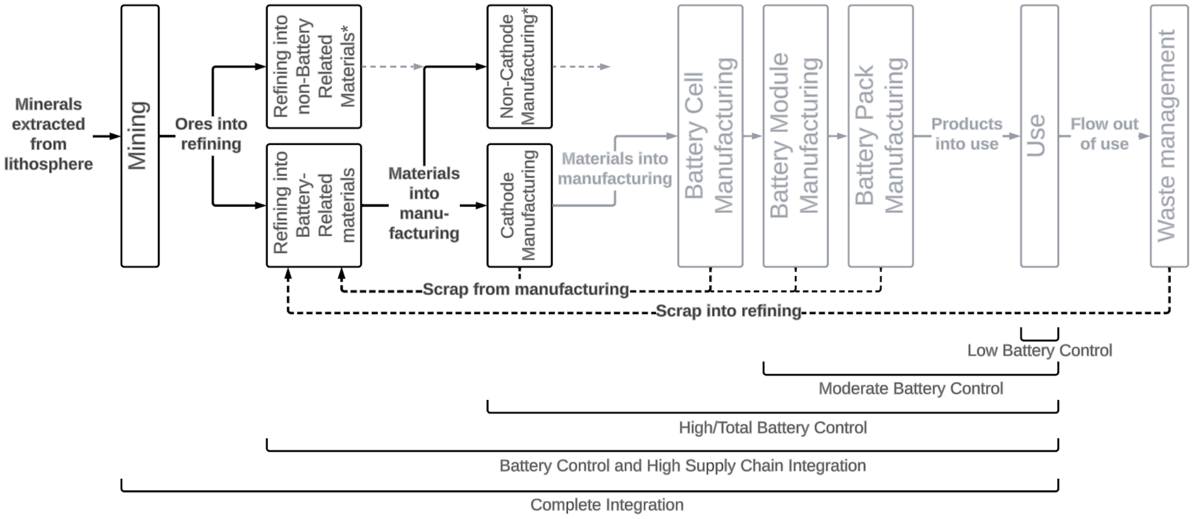

Supplementary Fig. 2. The simplified supply chain flows for battery materials considered in this study (in solid or dashed black lines), with manufacturer firm boundaries as described in Supplementary Table 1.

Note the steps in light gray are not modeled in this analysis; and the portions of the supply chain relating to non-battery related materials(*) would include any stockpiling of materials or manufacturing losses.

Supplementary Table 1. EV manufacturer decision making in their supply chain. Arrows indicate general movement of firms towards increasing direct control over their supply chains. Adapted and augmented from[7,8] and company press releases

**Supply Chain Control**	**Examples**
**Low Battery control:** Cell, module, and pack outsourced to 3rd party supplier, usually a battery manufacturer (e.g. CATL)	Many EV startups: NIO, Lucid, Fisker, etc.
**Moderate Battery control:** Cell production outsourced. In-house module and pack design and manufacturing	BMW, Renault, Daimler, VW↓
**High Battery control:** Cell production through joint ventures/ partnerships, and/or in-house module and pack design and manufacturing	Nissan, Mitsubishi, PSA (Stellantis), Toyota, Geely / Volvo, GM↓
**Total Battery control:** Firm manufacturing of cell, module, and pack design	BYD↓, Tesla↓, Ford↓
**Battery control and high supply chain integration:** Firm raw materials processing	
**Battery control and complete integration:** Integrated mining and raw materials extraction	

By definition, all companies producing their own battery cells, either in-house or through joint ventures or partnerships, are actively deciding the battery chemistries they have in their vehicle batteries, and thus where they source their battery cathode material from and the risks they might face due to global trade. Additionally, some companies (e.g. Ford,[9] GM,[10] Tesla[11]) have signed agreements with raw materials producers to supply their joint ventures and/or in-firm production, but may or may not directly control the exact sources of these materials, which may slightly muddy the clear delineations described in the table above. Some companies with lesser control may still choose to source batteries specifically because of the battery chemistry, but since they have no direct control, they also have less direct control over their exposure to risk from the upstream material supply chains.

Supplementary Text S1-2. Mineral Criticality Discussion and Other Trade-offs in Battery Design Choices

We analyzed the current vulnerability of the supply chains for four primary battery cathode materials: lithium, nickel, cobalt, and manganese, which are often considered as the primary 'critical' elements for EV batteries.[12–14] However, other materials that are sometimes considered 'critical' can be used cathode materials and/or batteries writ large, such as graphite, aluminum, phosphorus, copper, and fluorine. We summarize various perspectives on mineral criticality in Table A2-1, with a more general discussion of mineral criticality and how to measure it in Supplementary Text S2. The primary reason for this non- or less-critical categorization seems to be because the relative demand for such materials in batteries is small relative to the overall size of the market, and the number of countries that supply the material is high, as seen in Table A2-2. In general, if demand for the aforementioned minerals – or new minerals due to development of novel chemistries – becomes significant and/or are included on critical mineral lists, especially those related to batteries or energy technologies, then more detailed analysis would be useful in better understanding the vulnerabilities present in their supply chains. While we focus on Lithium, Cobalt, Nickel, and Manganese due to a general understanding that these are the most critical materials at the current moment and the fact that there exists sufficient data for these materials, likely due to their status as the most critical minerals, other materials that are not yet considered as critical typically have data issues, making it challenging to apply the full analysis method we have suggested here.

We take for example the mineral phosphorus, which in Table A2-1 is considered to be critical in some contexts, like other minerals excluded from the main body of the paper. Given that the criticality of phosphorus in the electric vehicle lithium ion battery context is uncertain, we provide a Sankey diagram in Supplementary Fig. 3, though data limitations prevent us from understanding country-level production of phosphoric acid that could be used as a precursor to LFP. We find demand for phosphorus for LFP production was 17,500 metric tons, as compared to the roughly 30 million metric tons of phosphorus mined in 2020 (223 Mt of phosphate rock)[15] - roughly 0.06% of total worldwide phosphorus demand. Even when narrowing down the phosphorus supply chain to phosphoric acid production, the primary precursor for the phosphorus used in LFP batteries,[16] this corresponds to roughly 0.075% of phosphoric acid demand. According to the USGS,[17] roughly 23.5 million tons of phosphate rock, or about 2.3

Supplementary Table 2. A review of the criticality of electric vehicle battery cathode materials.
Y: Yes, N: No, na: not applicable

	Li	Co	Ni	Mn	Al	P	Fe	Notes
[18]Xu et al. 2022 [EV Li-Ion]	**Y**	**Y**	**Y**	**Y**	na	N	na	Specifically responds to Spears et al.'s critique about the criticality of phosphorus.[15] Based on their analysis, they explicitly state that "we do not believe that phosphorus is as critical a raw material from a known reserves perspective as other battery elements…"
[19]Valero et al. 2021 [EV Li-Ion]	**Y**	**Y**	**Y**	**Y**	N	N*	N	*Phosphorus is only mentioned in the fact that phosphate rock is one of the six "most common minerals throughout the 20th century and beginning of the 21st century".
[20]Greenwood et al. 2021 [EV Li-Ion]	**Y**	**Y**	**Y**	**Y**	na	na	na	NMC specific analysis only.
[21]Ballinger et al. 2019 [EV Li-Ion]	**Y**	**Y**	**Y**	N	N	N	N	Describes 3 key elements "which are significant supply risks"
[22]Sun et al 2021 [Li-Ion]	**Y**	**Y**	**Y**	**Y**	na	na	na	Describes 15 commodities that include Li, Co, Ni, and Mn as the key LIB commodities that countries compete over
[23]Scott and Ireland 2020 [Li-Ion]	**Y**	**Y**	**Y**	na	na	na	na	Identifies four key LIB (these three plus graphite) materials based on various indicators (see Table 3).
[24]Matos et al 2020 [Li-Ion]	**Y**	**Y**	N	**Y**	na	na	na	Defines these three materials as "priority raw materials"
[13]Wentker et al. 2019 [Li-Ion]	**Y**	**Y**	**Y**	**Y**	N	**Y**	N	Primarily evaluated elements of supply risk and environmental risk. Phosphorus was identified as critical because of its low substitutability and high global supply concentration, being more critical than Mn but less than Li, Co, and Ni (see figures 2 and 3 in the paper)
[25]Sun et al 2019 [Li-Ion]	**Y**	**Y**	**Y**	**Y**	na	na	na	Identify these four materials as being "generally considered as the core elements for the LIBs"
[12]Helbig et al 2018 [Li-Ion]	**Y**	**Y**	**Y**	**Y**	**Y***	**Y**	**Y**	All are named as critical by construction; see Fig. 4 and 7. *Only Aluminum is notably 'less critical' in comparison.
[14]Olivetti et al 2017 [Li-Ion]	**Y**	**Y**	**Y**	**Y**	na	na	na	Focuses on two primary measurements: "static depletion index" and production concentration of top three countries
[26]Granholm 2021 [Energy Storage]	**Y**	**Y**	**Y**	**Y**	na	na	na	Specifically only calls out these four as critical
[27]Hund et al 2020 [Energy Storage]	**Y**	**Y**	**Y**	**Y**	**Y**	na	Y	See Table 3.1 "Energy Storage"
[28]Bauer et al. 2023 [Energy]	**Y**	**Y**	**Y**	N	**Y**	N	na	See Figure 5.1 in the paper. Phosphorus is assigned low supply risk (1-2) and low importance to energy (1) scores in both the short and medium terms, the lowest combined scores of any material analyzed with the methodology.
[29]USGS critical minerals list 2022	**Y**	**Y**	**Y**	**Y**	**Y**	N	na	The most general study, but this methodology is one of the standards for determining mineral criticality.

million metric tons of phosphorus were mined in the United States in 2020; it is clear the United States alone could supply the world's LFP-Phosphorus demand hundreds of times over. For further context, the USGS identified 71 billion metric tons of phosphate rock reserves (i.e. currently reasonably economically extractable material) around the world in 2020. While we recognize it is currently non-economical to convert most of these phosphorus resources (and indeed, nickel, cobalt, and manganese, to a certain extent) to the high level of quality and purity required for LFP batteries, in the vein of the Simon-Erlich wagers, we suggest that massive growth in market demand for such materials will encourage a shift in developing lower-grade resources for such applications and technological advancement to allow for such development. With the massive projected growth of all minerals due to the expansion of battery supply chains and the overall energy transition, we suggest that further efforts to gather better data and make such data available to researchers and analysts will be necessary to address future concerns.

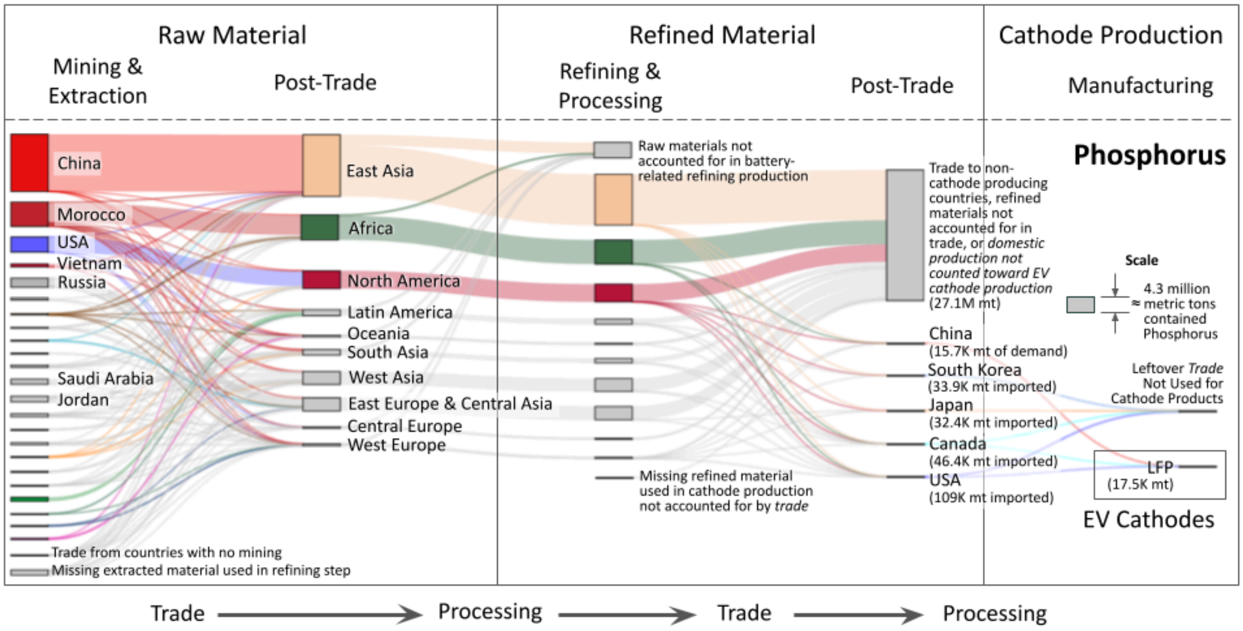

Supplementary Fig. 3. A Sankey diagram for global flows of phosphorus that available data suggest are involved in battery material supply chains.

See Supplementary Text S3-2 for further details and data sources. Note the scale of production - approximately 28 million tons of phosphorus (contained phosphorus) was extracted in 2020.

We note Supplementary Fig. 3 displays regional production data of phosphoric acid, the primary precursor used in LFP battery cathodes, as this level of aggregation was the most detailed that was publicly available.[30] As a result, we can only compare demand phosphorus used for LFP production to trade of phosphoric acid, and also cannot calculate a full vulnerability

Supplementary Table 3. Comparison of Production Geography and EV-related Material Demand, 2020[17,22]

**Material**	**Countries specifically listed in USGS Mineral Commodity Survey (MCS) [Number of countries]**	**Smallest amount produced by named country in MCS**	**Total Global Production**	**Material Demand for EVs**
Lithium	United States, Argentina, Australia, Brazil, Chile, China, Portugal, Zimbabwe [8]	348 metric tons (Portugal)* (*US production withheld)	82,500 metric tons	38,200 metric tons (46.3%)
Cobalt	United States, Australia, Canada, China, Dem. Rep. Congo, Cuba, Indonesia, Madagascar, Morocco, Papua New Guinea, Philippines, Russia [12]	600 metric tons (USA) (Second - Madagascar - 850 metric tons)	142,000 metric tons	87,800 metric tons (61.8%)
Nickel	United States, Australia, Brazil, Canada, China, Indonesia, France (New Caledonia), Philippines, Russia [9]	16700 metric tons (USA) (Second - Brazil - 77100 metric tons)	2,510,000 metric tons	145,800 metric tons (5.8%)
Manganese	Australia, Brazil, Burma, China, Côte d'Ivoire, Gabon, Georgia, Ghana, India, Kazakhstan, Malaysia, Mexico, South Africa, Ukraine, Vietnam [16]	121,000 metric tons (Vietnam)	18,900,000 metric tons	74,200 metric tons (0.3% of Mn demand)
Aluminum (Alumina)	United States, Australia, Brazil, Canada, China, Germany, Guinea, India, Indonesia, Ireland, Jamaica, Kazakhstan, Russia, Saudi Arabia, Spain, Ukraine, United Arab Emirates, Vietnam [18]	439,000 metric dry tons (Guinea)	136,000,000 metric tons	2,730 metric tons (5.2e-04% of raw Al production and 4.2e-03% of smelted Al production)
Aluminum (Bauxite)	United States, Australia, Brazil, China, Guinea, India, Indonesia, Jamaica, Kazakhstan, Russia, Saudi Arabia, Vietnam [12]	3,500,000 metric dry tons (Vietnam)	391,000,000 metric tons	
Aluminum (smelted)	United States, Australia, Bahrain, Canada, China, Iceland, India, Norway, Russia, United Arab Emirates [10]	860,000 metric tons (Iceland)	65,100,000 metric tons	
Phosphorus (Phosphate Rock)	United States, Algeria, Australia, Brazil, China, Egypt, Finland, India, Israel, Jordan, Kazakhstan, Mexico, Morocco, Peru, Russia, Saudi Arabia, Senegal, South Africa, Togo, Tunisia, Turkey, Uzbekistan, Vietnam [23]	577,000 metric tons (Mexico) ≈ 74,000 metric tons contained P	219,000,000 metric tons ≈ 28,000,000 metric tons contained P	17,500 metric tons contained P (0.06% of P demand)

index in the style of the other battery materials considered in this article. If we were to assume all production of refined phosphorus and phosphoric in East Asia occurs in China (which is approximately true based on discussion with representatives of the International Fertilizer Association), and only look at observed import data of those materials (rather than including domestic production), we can apply the methodology and see the results found in Supplementary

Table 4. We see that the measured vulnerability index values are not altogether that different from that of Lithium for LFP: slightly lower in the proportional case and going up to 100% in the 'known trade' pessimistic case. However, we cannot be certain of any measurements beyond the observed production of LFP in the cathode step, as we only observe inter-regional trade and thus cannot be sure if this is actually the vulnerability index for phosphorus and China.

Supplementary Table 4. China-based Vulnerability Index Calculations for lithium and phosphorus in the LFP supply chain.

*Indicates that this number is possibly a slight overestimate, as the middle stage of refining production only has regional data and we have assigned all production in "East Asia" to China.

	LFP - Li	LFP - P*
Optimistic Case (Minimum)	89.9%	89.9%
Base Case (Proportional)	91.6%	90.5%*
Pessimistic Case A (Known trade only)	93.2%	100%*
Pessimistic Case B (Extreme bound)	100%	100%*

It is important to acknowledge that the risks and vulnerabilities associated with each of our four chosen critical minerals varies. In the context of Lithium, for example, concerns have arisen over water extraction in South America and indigenous land rights in North America. Cobalt has been associated with child labor and heavy environmental damage in the Democratic Republic of the Congo.[14,31] Thus, while we simplify calculations of vulnerability to one dimension of supply availability, we recognize that tradeoffs exist in multiple dimensions across these four materials.

Lithium ion batteries themselves are composed of four primary components: a lithium compound cathode, graphitic carbon anode, liquid electrolyte, and polymer-based separator, as well as various other structural elements (e.g. copper and/or aluminum current collectors). There are some possible alternatives for electrolyzers, separators, and anode materials, but the diversity of choices in these are much fewer (e.g. anodes are currently made with graphite, with only some possibility for partial substitution with silicon),[32] so the opportunity for design alternatives is highly limited.

Few other battery cathode material alternatives exist for rechargeable batteries that have both sufficient energy density and power density at low enough cost, with the sodium-ion batteries being the only prominent non-lithium based cathode. Other traditional battery chemistries (e.g. Lead Acid, Zinc-Manganese Dioxide, Nickel Cadmium, Nickel Metal Hydride) are either non-rechargeable or not sufficiently energy dense. While relatively exotic chemistries like Lithium Metal, Lithium-Sulfur, and Lithium-Air batteries have been in various stages of development, they currently are not deployed for electric vehicles.[32–34] While Sodium Ion batteries have been developed for electric vehicles in China,[35] market penetration is currently low and the cathode materials often contain some mix of nickel, cobalt, and/or manganese, as well as other minerals such as titanium or copper, which does not fully remove the risks discussed in this article.

Beyond the critical materials required for each chemistry, other trade-offs exist – LFP battery pack energy densities are anywhere from 50 to 85% of that of NMC battery packs[33,36,37] while being 105% to 85% of the cost of production.[13,38] Thus, to achieve a battery with a certain amount of energy, differing amounts of critical materials would be required to build a battery of each type.

Supplementary Text S2. Literature Review

Supplementary Table 5. A summary of existing key literature on EV battery material supply chain vulnerabilities. We recognize that studies with these methods and themes could be used in the context of EV battery materials, but in some cases have not been applied in such a manner.

Literature stream	Use of countries as unit of geographic resolution	Explicit consideration of cathode material production	Quantifies flows of battery-related materials	Scope of studies includes multiple materials	Metrics of merit for quantifying relative importance of materials	Characterizes uncertainty	Units used	Example studies
Energy security	Often	No (not in scope)	N/A	N/A	N/A	N/A	N/A	[39–42]
Materials criticality	Often	Small number of studies	No	Often	Yes, various indices	Yes	Mass, indices	[12,19–21,27,43,44]
Material flow analysis	Often	Small number of studies	Yes	Unusual, usually one material	No, focus on mapping flows	Unusual, usually a ‘snapshot’	$, mass	[1–3,45–47]
Input-output analysis	Sometimes, sometimes subnational or regional in scope	Could be used for this, but no related studies explicitly do	Yes, but with proportionality assumption	Sometimes, usually considers materials in one industry	No related studies	Often	$, mass	No Li-ion battery material studies, some battery-related work[48–50]
Supply chain disruption propagation	Often	Not explicitly found in these studies	Yes, but with proportionality assumption	No, focus on single materials	No, calculation of number of countries affected by disruption	Yes, by varying probabilities of disruption	Mass	[51–54]
This study	Yes	Yes	Yes	Yes	Yes, relative vulnerability	Yes, via bounding analysis on missing data	Mass	–

To answer our research question, we need to build a model that maps the battery material supply chains at a national level and measures interactions between trade flows and production capacity, while accounting for uncertainty and missing data. Several streams of literature have investigated relevant concepts and metrics, including literature on energy security, materials criticality, material flow analysis, input-output analysis, and supply chain disruption propagation. The relationship between our model and these literature streams is summarized in Supplementary Table 5.

Energy security debates have long focused on where energy resources are sourced, with definitions often made in terms of access to fuels or security of supply, largely in the context of oil and other fossil resources.[55,56] Numerous studies of past and potential disruptions of oil supply

and the threat of the 'oil weapon' have considered specific regions and countries.[39–42,57] However, transitions to renewable and electricity-based systems have introduced new geographical dependencies that differ from those raised by fossil fuels.[44,58,59] The materials criticality literature was in part developed to address these nuances.

The literature on materials criticality attempts to assess the relative risks and vulnerabilities associated with the supply of minerals and materials. These assessments often aggregate geological, technological, and economic measurements with social, regulatory, and geopolitical indicators into overarching summary metrics.[60–62] These aggregated metrics have been applied across multiple battery supply chains and battery chemistries, particularly from the perspective of import dependence, rather than the relative impact of various countries' involvement in supply chains. For example, some studies[12,20,43] measure concentration of production with the Herfindahl-Hirschman Index and then weight them (using indices such as the World Governance Indicators, material demand, import reliance, etc.) to account for different levels of risk. For example, the first of these studies[12] aggregates four measures of risk for battery-related critical minerals in the context of various cathode material compositions, finding that in a simple arithmetic mean aggregation, LFP is the only battery chemistry with "measurably lower supply risk compared to the other battery types." While these metrics can capture a broad range of concerns, they may mask the dimensions that are important for ensuring supply availability throughout a specific product's entire supply chain,[63,64] including obfuscating the vulnerability of risk caused by specific countries. In the case of battery materials, while we learn that lithium and cobalt tend to be 'more critical' than other battery minerals, we suggest that these measurements do not adequately describe the importance of specific countries and their trade inter-dependencies, especially in the context of battery material chemistry choices and their supply chains.

The material flow analysis literature maps and characterizes the production and flow of materials using international trade data and various sources of production data to better understand geographical relationships of supply chains. In the context of battery materials, some of this literature focuses on specific stages of the value chain, e.g. raw materials and mining, while others encompass all steps. Most of these trace specific materials at a global context, such as lithium,[1] cobalt,[2,46] nickel,[47] and manganese.[65] Additional studies have considered all of the cathode materials in a regional context,[24] though there is little explicit inclusion of the cathode

material production step in these material flows outside of one study.[1] One recent material flow analysis-adjacent study[66] evaluates the feasibility of meeting the recently passed IRA's goals for minimum critical mineral requirements across all battery materials for the United States, finding that achieving the market value-based target may be possible with NCA batteries but not necessarily for LFP or NMC batteries. We provide an analysis in the context of potential mass-based targets and identify a need for a global perspective. In general, while studies describing the state of supply chains are useful for describing the structure of material flows and supply chains, allowing for analysis of potential vulnerabilities, none of these studies quantitatively measure the interdependencies between specific technology choices and material mass requirements on specific countries.

Two types of studies have considered different ways to measure relationships between steps of supply chains between production geographies, though not necessarily in the specific definition of vulnerability we have used in this article. The input-output (I/O) literature measures flows of materials, emissions, and other quantities through interconnected industries, sometimes through the perspective of a specific material's or product's supply chain. These models can calculate measures of vulnerability through matrices of relationships between industries or steps of a supply chain, making the assumption that input proportions between each stage or step are fixed.[67] While input-output models are typically described in terms of one unit (e.g. dollars), a subset of this literature, known as a 'mixed-unit' input-output technique, have focused on combining I/O models with material flow analysis, using mass balance to measure physical flows of materials and sometimes energy or emissions.[68] These studies track material flows in detail across steps of the supply chain, but they rely on proportionality constants and thus can only provide point-estimate snapshots of vulnerability on certain geographies. Furthermore, there has been little study of battery material supply chains with input-output analysis, with existing studies focusing on either just lithium-ion battery manufacturing[48] or in non-lithium-ion contexts.[49,50]

Other studies have focused on 'supply chain disruption propagation' for certain battery materials, such as natural graphite,[53] cobalt ores,[54] lithium carbonate and lithium batteries,[51] and nickel.[52] These studies calculate the 'avalanche size' of a disruption – the number of countries affected by the removal of supply or trade from one country – while assuming that disruptions occur as proportional losses of trade from the disrupted country that are propagated to other

countries. Most of these studies underscore the significance of China as a critical node in the supply chain though these studies are limited to specific trade codes or materials. Furthermore, all of these studies except the study on nickel do not consider the fact that there are relationships between countries at multiple steps of the supply chain, and the nickel study uses a correlation instead of considering the actual physical quantities of materials that are converted from one step of the supply chain to the next. While this measure of vulnerability may be helpful in understanding linkages between countries, we suggest that the lack of analysis across multiple materials and the physical quantities of materials needed constitutes a gap in the literature.

As summarized in Supplementary Table 5, no existing models incorporate these six primary qualities, so we create a new model, leveraging prior work and bridging these gaps, to answer this question. We work backwards from lithium ion battery cathode material requirements to map the entire supply chain on a country-by-country basis, with awareness of the specific amount of constituent materials needed for each cathode material. We measure overall vulnerability on any given country, going beyond the proportional measurements found in the input-output and supply chain disruption propagation literatures by bounding the possible range of disruption on any given region of interest in the supply chain.

Supplementary Text S3. Full Methodology and Data

Supplementary Text S3-1. Methods

Data Aggregation

We model the supply chain for each chemistry by considering supply of each material for each battery chemistry, focusing on mining, refining, and cathode material production. Each country can produce, import, and export material at multiple steps in the supply chain, and thus the portion of end cathode material that involves a given country in the supply chain varies depending on the amount of end material being produced and the trade relationships between all countries.

While the supply chain for each material can be complex, we make a number of simplifying assumptions given the data available (see Supplementary Fig. 1 and Supplementary Fig. 2). We do not incorporate stages after battery cathode material manufacturing, but include the movement of scrap, with the caveat that little of it is currently involved in the electric vehicle battery supply chain (most nickel and manganese scrap is used in the iron and steel market,[24,65,69] while cobalt scrap is largely from superalloys[46,70]). Additionally, we generally make the assumption that no firm-level buffers/national stockpiles of these materials exist or are minimal in size relative to trade and/or production.

We aim to represent the flow of materials via trade and processing as a two-dimensional network. We do this by assuming that countries with known production are the only countries with the technological production capability to produce materials at each step, and that no one else has this capability and thus must trade from these producers in order to get these materials.

We track five battery material supply chains: the lithium in LFP cathode materials, and the lithium, nickel, cobalt, and manganese in NMC cathode materials. For each step of the supply chain, we compute a mass balance:

$$D_{mi} + \sum_{j \in J} T_{mij} = S_{mi} + \sum_{j \in J} T_{mji} \; \forall m \in M, \forall i \in J$$

Supplementary Equation 1. Total Demand (Demand+Exports) = Total Supply (Production+Imports), for all materials and countries

where D_{mi} is the mass of material m used to make all potential products in country i, T_{mij} is the amount of material m traded from country i to country j, J is the set of all countries, and S_{mi} is the mass of material m produced in country i. This equation holds for each material $m\in M=\{\text{Li,Ni,Co,Mn}\}$ and each country $i\in J$.

Total trade between countries may involve some trade that is observable and some trade that is unobservable due to smuggling, unintentional misclassification, tariff avoidance, and other reasons.[3,46,71] We represent these as follows:

$$\sum_{j\in J} T_{mji} = \left(\sum_{j\in J_{prod}} T^{OBS}_{mji} - \sum_{j\in J\setminus J_{prod}} T^{OBS}_{mji}\right) + T^{UNOBS}_{mji} \; \forall m \in M, \forall i \in J$$

Supplementary Equation 2. Total Imports = Imports from Producing Countries + Imports from Non-Producing Countries + Unobserved Imports, for all materials and countries

where T^{OBS}_{mji} is the observed trade from country j to country i available in the data, J^{PROD}_{m} is the subset of countries that are known to produce material m, and T^{UNOBS}_{mi} is unobserved trade of material m to country i that is missing in the data. We compute the net value of T^{UNOBS}_{mi} (positive or negative) needed to satisfy Supplementary Equation 1 for each material, country pair, effectively assuming that any mismatch between supply and demand in the data is explained by unobserved trade. The countries that export relevant materials but are not known to have production may obfuscate the true 'original source' of the material, assuming the countries listed with production capacity are the only ones that have the technological capability. This creates additional uncertainty.

$$D_{mi} = \sum_{p\in P} d_{pi} \cdot c_{pm}$$

Supplementary Equation 3. Total material D demanded = Supply d of product p ✕ material needed per product

$$S_{mi} = \sum_{\alpha\in A} s_{\alpha i} \cdot c_{\alpha m}$$

Supplementary Equation 4. Supply of contained material S = Supply s of compound supplied α ✕ contained material per compound

$$T_{mji} = \sum_{\beta \in B} t_{\beta ji} \cdot c_{\beta m}$$

Supplementary Equation 5. Trade T between countries *j* and *i* = Trade *t* of items β × contained material per item

Furthermore, we cannot directly observe the movement of material *m* in its pure state (e.g. contained nickel compared to nickel ores and concentrates, nickel mattes, nickel sulfates, unwrought nickel alloys, etc.). Assuming no losses in the production processes, we use conversion factors *c* to convert from input materials α with supply $s_{\alpha i}$, imported goods β at amount $t_{\beta ji}$, and demand from produced products d_{ip} to their contained material amounts, S_{mi}, T_{mji}, and D_{mip}, as seen in Supplementary Equations 3 through 5. These conversion factors *c* are described below in the *Data Sources* section (Supplementary Text S3-2) and compiled with associated trade codes in Supplementary Text S3-3. Values for $t_{\beta ji}$ are associated with relevant trade codes in Supplementary Text S3-3.

As a result, combining all unobserved trade into one variable and trade from and to non-producing countries into another, recognizing that quantity can be positive or negative, we describe our total supply-demand balance in Supplementary Equation 6.

$$\sum_{p \in P} d_{ip} \cdot c_{pm} = \sum_{\alpha \in A} s_{\alpha i} \cdot c_{\alpha m} + \sum_{\beta \in B} \left(\left(\sum_{j \in J_{prod}} t^{OBS}_{\beta ji} \right) - \left(\sum_{j \in J \setminus J_{prod}} t^{OBS}_{\beta ij} \right) \right) \cdot c_{\beta m} + T^{UNOBS}_{mi}$$

Supplementary Equation 6. Total demand for each material = Material supply + (Material imported – Material exported) + Unobserved Trade

We can measure the variables in Supplementary Equation 6 in terms of demand for cathode materials that require refined minerals. These refined minerals in turn require raw minerals, so we have a two step process, where the total supply of refined materials S_{mi} in the refining-cathode material flow analysis is a subset of the products considered in the total demand for refined materials D_{mip} in the mining-refining material flow analysis.

Vulnerability Analysis and Uncertainty Bounds

We calculate the total vulnerability of cathode material supply for a set of countries, which we designate as the countries of focus. Clearly, any cathode production in these countries would count towards vulnerability due to those countries. However, other countries that produce

cathode material could potentially also be vulnerable to the countries of focus for refined materials, while the countries that supply those cathode material producing countries with refined materials could also be dependent on the countries of focus for raw materials. Taking into account this 'ripple effect' across multiple supply flows, we determine the quantity of cathode material dependent on our countries of focus across cathode material production, materials refining, and raw materials production.

To bound this analysis, we consider both uncertainty due to *observability of trade* between countries that goes to battery end-products, and uncertainty due to *unobserved trade data*: trade that should exist in order to produce battery products but otherwise is unobserved in the trade data. Using these, we measure the amount of vulnerability based on a country in four ways.

In a **proportional trade** case, we find the total amount of internal supply less exports, and the total amount of imports for each country with production capability at each step. With this data, we can calculate the percentage of supply at each step that depends on the countries of focus. By aggregating these percentages at each stage of production, we can calculate an overall percentage of the end cathode production that becomes unavailable. In this scenario, we assume any unobserved trade follows the same patterns as observed trade. This follows methodologies described in input-output and supply chain disruption propagation literature.[49,51–54,67,68,72–74]

The other three vulnerability calculations use the concept of maximum flow[75] to calculate the minimum and maximum vulnerability on any set of focus countries. We translate our Sankey diagrams into network flows. Nodes of the network correspond to the vertical bars in each Sankey diagram at each of the five stages of the diagram – mining, mining post-trade, refining, refining post-trade, and cathode production, while connections in the network correspond to the flows in each Sankey diagram between the stages, representing either trade (internal or international) or material processing between each stage. Furthermore, we add a 'supersource' node where all mined and raw material originates from, with capacity equal to the amount of production claimed by each raw material producing country.

In a **maximum known trade** case, we try to optimize the flow of material across our network, such that as much of it passes through the countries of focus as possible. By connecting the supersource node to all of the countries of focus at each step of the supply chain, with effectively infinite capacity in these flows, we can apply a maximum flow algorithm (in this case

the Edmonds-Karp algorithm[76]) to calculate the maximum vulnerability based on the countries of focus. Through careful analysis of the distribution of flows using the outputs of the algorithm, we can determine the vulnerability at each step of the supply chain.

To understand the impact of **unobserved trade in the maximum trade** case, we assume it could all come from the countries of focus. In practice, we connect the nodes that represent uncertain supply (MMRS, TCNM, UARP, TCNR, MRMC) to the supersource, to include them as potential sources of material. However, not all of these materials can necessarily come from our country of focus if they are not actually known to produce materials in the steps upstream of the uncertain supply, e.g. Russia is not known to be a producer of raw or refined lithium, so none of the uncertain lithium supply should come from Russia. Thus, we include the (still relatively naive) assumption that the maximum flow from our supersource to any of these uncertain supply nodes cannot exceed the sum of the production across all countries of focus at the step that the uncertain supply node joins the material flow network.

Supplementary Table 6. Flow Constraints in the maximum trade case that includes uncertain (unobserved and indirect) trade data

Included uncertain data source	Flow Constraint	Reasoning
MMRS (Missing (extracted) material used in refining step) *plus* TCNM (Trade from countries with no mining)	Amount countries of focus that is TCNB (Trade to countries with no battery-related refining, or raw materials not accounted for in trade) *plus* amount countries of focus that have RMRP (Raw materials not accounted for in battery-related refining production)	TCNB and RMRP are our materials in raw form that could have been re-imported or miscategorized / misrepresented as not feeding into the next step.
UARP (Unaccounted-for additional refining production) *plus* TCNR (Trade from countries with no refining) *plus* MRMC (Missing refined material used in cathode production)	TCNB + RMRP *plus* the amount countries of focus that is TNPC (Trade to non-cathode producing countries, or refined materials not accounted for in trade) or assigned as Non-Cathode Products	We assume any of the TCNB and RMRP could have been converted at some point.<br>TNPC is any ‘missing’ refined materials, while any refined material is not recorded as being used in cathode material manufacturing could have been re-imported or

		miscategorized

A **minimum trade** case is the same measurement as the pessimistic scenario, but instead chooses the countries that import the least quantities of materials, and further assumes unobserved trade does not come from the country at all (minimal vulnerability based on the country in question). The practical implementation of this is to calculate the inverse – determining the maximum flow through the network where all flows into and out of nodes in the countries of focus are not included in the network, and subtracting that amount from the expected demand at the last (cathode-producing) step to determine the amount of material that at minimum must come from the countries of focus.

Supplementary Text S3-2. Data Sources

Production Data ($s_{\alpha i}$ and d_{pi})

We choose to use 2020 USGS mining data[17] like many other studies in the critical materials and material flow analysis literature.[14,25,46] These data are largely reported in terms of contained minerals (e.g. metric tons of lithium equivalent or thousands of tons of nickel equivalent), which reduces uncertainty from needing to use conversion factors, as described below. While there may be some lag in production processes, such as lithium brines requiring up to a year to process via evaporation,[6] USGS production data is reported in terms of final production quantities (e.g. after lithium concentration). We use refined materials and cathode materials production data (as well as US lithium production) reported in Sun et al. 2021,[22] which is largely from 2020. For the analysis of phosphorus included in Supplementary Text S1, given that granular production data for the refining step (phosphoric acid) was not found, we used regional production statistics from IFASTAT, the International Fertilizer Association's statistics website.[30]

Trade Data ($t^{OBS}_{\beta ij}$)

Following methodologies from the material flow analysis literature,[1–4,23,46,47,65,69,70,77–79] we trace inter-country trade from country to country by identifying relevant Harmonized System (HS) codes. These codes, internationally standardized by the World Customs Organization to 6 digits, allow for importing countries to levy tariffs and monitor compliance with regulations (e.g.

rules of origin), and as such are designed to classify and cover all internationally traded items.[80] Most sources of trade data are ultimately based off of the UN Comtrade database, which aggregates reports submitted by UN member countries on their trade at a high level, which is the database used by nearly all global material flow analysis and supply chain disruption propagation studies reviewed in this article.[2–4,23,25,46,47,51–54,65,70,74,77–79] Other national and regional-level trade databases, such as the US Census's database or Eurostat, are much more detailed, but do not cover the entirety of trade around the world, and as such are less useful when considering whole supply chains. These national and regional databases could be used to supplement data gathered from international sources, which is what is done in the data source we use. It is also important to note that both export and import data exist in these databases. Nearly all reviewed studies in the material flow analysis and supply chain disruption propagation literatures that specified a choice between the two used import data[2,3,46,51,54] rather than export data,[65] as it tends to be more complete and accurate, as many countries impose various import tariffs and are concerned about what materials enter their borders,[2,46,80,81] so we also use reported import data figures.

We use **TradeMap** data from IntraCen[80] as it provides the UN Comtrade data while additionally supplementing it with national and regional trade where import and export data may be conflicting or missing, as some countries do not report data to UN Comtrade. It is the opinion of the authors that this incorporation of national and regional trade data positively augments the UN Comtrade database upon which most material flow analyses base their analyses on. We use 2020 data to synchronize with our 2020 production data, and as a result rely on the 2017 HS code nomenclature. Databases based on the UN Comtrade dataset report their data in both dollar value and quantities. While using either has its drawbacks (commodities have constantly fluctuating prices and materials imported under trade codes are not homogeneous and not all shipments are accurately weighed and contained amounts of the critical material vary), we chose to use the latter to minimize the impact of uncertainty and issues of propagated errors. This necessitates the use of *Conversion Factors* (as described in the next subsection) to change the units of the broad categories of each trade stream into terms of contained materials. 28 HS codes were selected to represent trade of materials at each stage of the supply chain between mining and cathode manufacturing (see Supplementary Table 7). We reference a variety of literature to fully aggregate across all trade codes that may include battery material supply.[2,20,22,23,25,46,77,82,83]

We make the assumption that the traded raw and refined material in the selected codes is representative of materials that could actually end up in battery cathode materials. Globally, about 74% of mined lithium[17] and 57% of mined cobalt[84] is used in lithium ion batteries, but only a portion of lithium ion batteries are used in electric vehicles. Furthermore, only about 11% of nickel[85] and 2% of manganese[24] is used to make batteries of any kind. Different grades of ores, refining processes, and precursor materials, including recycled and scrap materials, make it more economically efficient to produce certain types of materials, not all of which can be used for battery cathode material production, and not all battery cathode materials are used in electric vehicles. However, as it is *possible* to use any of these materials in the electric vehicle battery material supply chain with enough processing, we choose to include all of these trade data in our analysis. The relevant supply chain flows included in our dataset are found in Supplementary Text S1-1 and Supplementary Fig. 2. Additionally, while other studies account for missing trade by exploring country-specific trade codes and known inter-country trade relationships, other than the export of Australian lithium, we do not account for any of these potential clarifications of the missing data problem.

We recognize that this snapshot of a dynamic system is likely not to be perfectly accurate; our inclusion of uncertainty analysis around our primary point estimates attempts to account for these challenges. Additionally, with improved data, we suggest the power of this methodology only increases.

Conversion Factors ($c_{\beta m}$)

In order to map the flows of each material in terms of the actual amounts traded, rather than the numerous compounds containing various amounts of the material, conversion factors are used to standardize all trade in terms of units of contained material. We reference a number of sources for estimates[1,4,22,47,65,86,87] for these coefficients for each trade code (see Supplementary Table 7). We recognize that these are point estimates and therefore possibly uncertain and/or are affected by anchoring bias; the few sources with overlapping trade codes generally agree on these conversion factors (or cite these original studies to begin with).

Supplementary Text S3-3. Trade Codes and Conversion Factors

Supplementary Table 7. Trade Codes and Conversion Factors used in this analysis

The codes and conversion factors in **bold** below are the ones included in the analysis. Explanations for the exclusion of codes are noted in the corresponding labeled table notes. S: Self-calculated or estimated

	Code	Name	Source	Conv.	Source
Raw lithium	**253090**	**Lithium concentrates** [a]	[77]	**0.03**	[4]
Refined lithium	280519	Alkali and alkali earth [b]	[77]	0.03	[4]
	**282520**	**Lithium oxide and hydroxide**	[20,22,77]	**0.165**	[4], **S**
	282690	Fluorosilicates of sodium or of potassium [b]	[77]	-	-
	282739	Lithium Chlorides [b]	[77]	0.164	[4], S
	**283691**	**Lithium carbonates**	[20,22,77,83]	**0.188**	[4], **S**
Raw nickel	**260400**	**Nickel ores and concentrates**	[20,22,79,82,83]	**0.015**	[47]
Refined nickel	**282540**	**Nickel oxides and hydroxides**	[20,25,82]	**0.787**	[47]
	**282735**	**Nickel chlorides**	[25]	**0.454**	[47]
	**283324**	**Sulphates of nickel**	[20,22,82,83]	**0.224**	[47]
	720421	Ferrous waste & scrap of stainless steel [c]	[25]	0.024	[47]
	**750110**	**Nickel; nickel mattes**	[20,25,82]	**0.75**	[47]
	**750120**	**Nickel oxide sinters**	[20,25,82]	**0.75**	[47]
	**750210**	**Nickel, not alloyed, unwrought**	[23,82]	**0.995**	[47]
	**750220**	**Unwrought nickel alloys**	[23,82]	**0.5**	[47]
	**750300**	**Nickel; waste and scrap**	[25,82]	**0.5**	[47]
	**750400**	**Powders and flakes, of nickel (excluding nickel oxide sinters)**	[23,82]	**0.995**	[47]
Raw cobalt	**260400**	**Nickel ores and concentrates**	[2]	**0.001**	[2]
	**260500**	**Cobalt ores and concentrates**	[2,20,22,46,77,79]	**0.15**	[2]
Refined cobalt	**282200**	**Cobalt oxides and hydroxides; commercial cobalt oxides**	[2,20,22,46,77,79,83]	**0.329**	[2]
	282734	Cobalt chlorides [d]	[2,22]	0.454	[2]
	**283329**	**Sulphates**	[2]	**0.01**	**S**
	283699	Carbonates, Others; Peroxocarbonates (percarbonates) [e]	[2]	-	-
	284190	Other salts of oxometallic or peroxometallic acids [e]	[2]	-	-
	810510	Cobalt mattes and other intermediate products of cobalt metallurgy [f]	[2,20,46]	-	-
	**810520**	**Cobalt mattes and other intermediate products of cobalt metallurgy; unwrought cobalt; cobalt powders**	[2,22,46,77,79]	**0.6**	[2,46]
	**810530**	**Cobalt waste & scrap**	[2,22]	**0.6**	[2]

	**810590**	**Articles of cobalt, n.e.s.***	[2,22,46]	**0.6**	[2,46]
Raw manganese	**260200**	**Manganese ores and concentrates**	[20,22,82,83]	**0.3**	[65]
Refined manganese	**282010**	**Manganese Dioxide**	[20,22,82]	**0.632**	[65]
	**282090**	**Manganese oxides; excluding manganese dioxide**	[20,82]	**0.774**	[65]
	283321	Magnesium sulfate [g]	[83]	-	-
	**283329**	**Sulphates**	[83]	**0.02**	**S**
	**811100**	**Manganese; articles thereof including waste and scrap**	[82]	**0.004**	[65]
Raw Phosphorus	**2510**	**Phosphate, Ground or Unground**	[86,87]	**0.119**	[86,87] **[h]**
Refined Phosphorus	**280470**	**Phosphorus**	[86,87]	**1**	[86,87] **[h]**
	**280920**	**Phosphoric Acid**	[86,87]	**0.273**	[86,87] **[h]**
LFP	**N/A**			**0.0440**	**S [i]**
NMC	**N/A**			**0.0716**	**S [i]**

[a]: While most lithium is produced from brines that are refined into lithium hydroxide or lithium carbonate forms near the mining sites, lithium as an ore (which is currently essentially all found as spodumene) is presently only produced in Australia and China[17] and not tracked as a specific commodity in the HS system.[80] Noting that only Australia and China have any lithium spodumene ore production,[17] we assume all Chinese production is domestically consumed for refining, and refer to Australia's country-specific export data of "25309011 Lithium concentrates", using the "ores" conversion factor from Sun et al. 2018 to convert it to quantity of contained lithium, to estimate Australian lithium exports.

[b]: The codes 280519, 282690, 282739 are used in lithium-ion batteries, sometimes as lithium alloys, but primarily as inputs for battery electrolytes.[77] Because materials covered under these trade codes have an unclear relationship with cathode material production (and cathode materials are already traded under other trade codes), we disclude these from our trade analysis.

[c]: Discluded as it primarily refers to stainless steel waste and scrap

[d]: Discluded as it removed in 2007 HS code revision[80]

[e]: Discluded as unclear which of cobalt compounds listed would be included, and cobalt content is highly uncertain

[f]: Discluded as it removed in 2002 HS code revision[80]

[g]: This study[83] mistakes magnesium for manganese and thus this is not included; this code is for magnesium sulfate[80]

[h]: Li et al.[87] identify the conversion factor for Phosphorus rock (ground or unground) to be 9.8%. Chen and Chen[86] assume 32% of the weight of phosphate rock is P_2O_5, which, using a chemical formula, results in about 14% of the rock as phosphorus. We take the average of these two numbers. We assume the phosphorus content in phosphorus is pure (100%), while for phosphoric acid, we take the average of the values of phosphorus in phosphoric acid calculated from chemical formula (0.316), Li et al. (0.268), and Chen and Chen (0.236).

[i]: Using chemical formulas for LFP ($LiFePO_4$) and NMC622 ($LiNi_xMn_yCo_zO_2$, $x+y+z \approx 1$), we find conversion factors of 0.0440 and 0.0716, respectively. Other sources[4] list them as 0.07 and 0.04, respectively, instead; it is possible that they were accidentally swapped in their analysis. We choose to use NMC622 as it is quite close to the average chemical composition based on a predicted market size-weighted blend of NMC technologies, as seen in Supplementary Table 8. This calculation is based on a simulated share of choices presented in a projected LFP-dominant scenario,[88] which is what is currently empirically happening as the electric vehicle market is developing.

Supplementary Table 8. NMC simulated share of choices and representative chemistry analysis.[88]

We bold NMC622 and the Weighted Average rows to highlight their similarity; we use NMC622 as the rough approximation of the average NMC chemistry

	Molecular Weight	Chemistry	Ni	Co	Mn	Total Mole. Weight	%Li	%Ni	%Co	%Mn	Predicted Share
Lithium	6.941	NMC333	0.333	0.333	0.333	96.46	7.2%	20.3%	20.4%	19.0%	4.1%
Nickel	58.6934	NMC523	0.500	0.200	0.300	96.55	7.2%	30.4%	12.2%	17.1%	10.7%
Cobalt	58.9332	**NMC622**	**0.600**	**0.200**	**0.200**	**96.93**	**7.2%**	**36.3%**	**12.2%**	**11.3%**	12.2%
Manganese	54.938	NMC721	0.778	0.222	0.111	103.79	6.7%	44.0%	12/6%	5.9%	0%
Oxygen	15.9994	NMC811	0.800	0.100	0.100	97.28	7.1%	48.3%	6.1%	5.6%	2.9%
Iron	55.845										
Phosphate	30.9738	**Weighted Ave.**	**0.547**	**0.209**	**0.244**	**96.77**	**7.1%**	**33.2%**	**12.7%**	**13.9%**	

Supplementary Text S3-4. Qualitative Discussion of Limitations

We recognize that this analysis has a number of limitations. We only consider a first-order measurement of vulnerability based on specific countries or sets of countries, rather than considering a more realistic analysis where a disruption occurs, such as where a country's production partially shuts down, or trade in a specific material is restricted. This analysis does not consider any market or government responses, in the form of supply increases, price shocks, contracts (either upheld or broken), or drawing upon stockpiles. Furthermore, we recognize that a static snapshot of the market does not necessarily tell us how the market may evolve as additional production capacity comes online and different policies and restrictions affect trade. We do note that most countries have very limited amounts of refined and cathode materials stockpiled, and that it takes 5-15+ years to prospect, plan, fund, permit and deploy a new mining site[6] and about 7 years to build a mine and refining plant,[89] so market responses to increase supply outside of using any possible slack supply will not occur in the short term. In general, market reports seem to indicate very little to no slack capacity in materials refining and processing and cathode manufacturing plans.

We caution against using these figures as exact measurements, given the age of the data (2020) and the number of assumptions needed to be made about the type of data. For example, because NCA and NMC were not separated in our source data, we make an assumption about the relative market shares of each based on projected market shares,[88] and assume production is homogeneous across China, Japan, and South Korea, the three NCX manufacturers. While we recognize that these numerical estimates are from 2020 data, which may not necessarily be representative of trade in a "business as usual" scenario given the COVID-19 pandemic and subsequent restrictions in global trade, we do argue that the rapidly developing nature of this industry means the most recent available data is most appropriate for characterizing a snapshot of potential risk. Furthermore, several projections indicate even higher potential future reliance on China in the refining and cathode production steps given current growth trends,[90] such as its share of overall cathode production increasing from 78% to 87% by 2030,[91] as well as Russia, DRC, and South Africa in the raw material production steps, so the result with the least missing data uncertainty may still be underestimating the amount of current and future risk. Of course, this methodology could be extended to analyze the change in vulnerability over multiple years or

smaller time-frames, assuming the ability to gather both production and trade data within the time interval in question.

This analysis could also be extended with further awareness of country-based ownership of production capability, given data about production quantities by individual firms, which currently has not been aggregated for all materials in the battery material supply chain. The fact that other countries' companies control assets in other countries may result in different risk factors that these vulnerabilities create. While these issues of firm choice and control may be less 'strict' bounds on market dynamics, these firms likely could maintain some market power in choosing the customers they sell to.

This work begs the question about market responses as the markets grow and evolve. These trade relations are not static (though long-term supply contracts are potentially significant), and neither are the production capabilities. We recognize that market structures can and have changed in response to economic factors (price shocks, etc.) and political disruptions (export bans, tariffs, etc.). Describing scenarios of disruption as well as understanding firm best responses to such disruptions and production displacement is a natural next step in this work.

Supplementary Text S4. Further Results

Supplementary Text S4-1. Additional Vulnerability Index Calculation Visualizations for NMC Cathode Supply, for Cobalt, Nickel, and Manganese supply

Note that, while the example solutions in the below figures and Fig. 4 show a significant involvement of uncertain flows in the minimum case, there exist other possible solutions, as the optimizer only presents one solution. There likely exist other solutions with less involvement of uncertain flows, but the optimizer was not coded to minimize use of uncertain flows. Additionally, note that sums may not add evenly due to rounding.

Table 1 in the main body of the paper defines the four sensitivity cases. The amount of overall vulnerability at the cathode manufacturing step, added at each upstream step, is noted with a plus (+) sign. The solid horizontal black lines are visual aids to indicate separation of countries; semi-transparent red bars represent vulnerability that is propagated downstream.

Indirect trade and unobserved trade do not count towards the measurement of vulnerability in the optimistic case, but are counted towards the pessimistic calculation in the case that includes uncertain data. For the minimum and maximum cases, one possible solution is presented, but other distributions are possible.

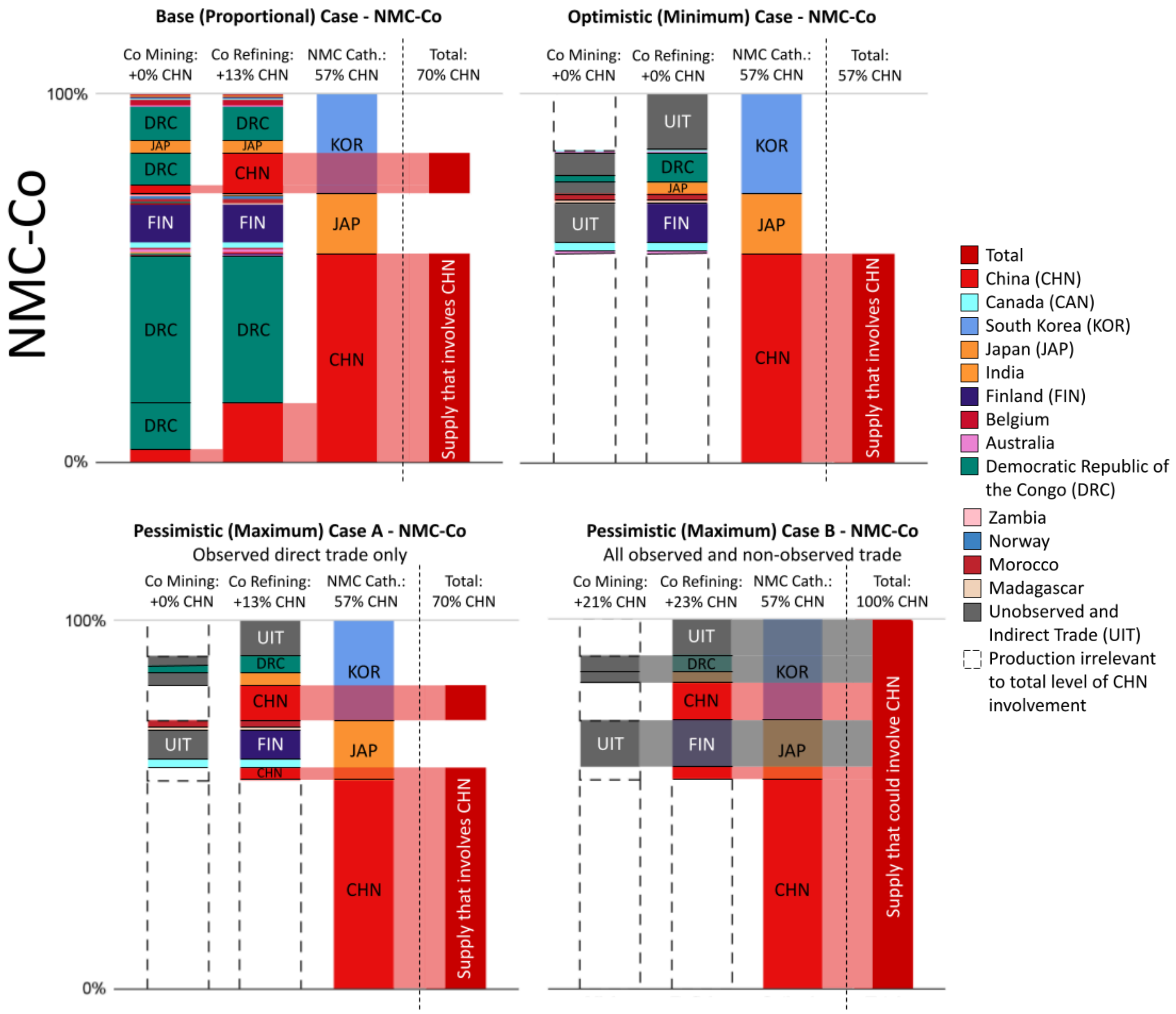

Supplementary Fig. 4. Visualization of a vulnerability index for the global NMC (Lithium Nickel Manganese Cobalt) cathode supply, for a cobalt supply chain disruption in China. Note that India, Belgium, Australia, Zambia, Norway, and Madagascar are represented on these diagrams, as identified in the color legend on the right hand side, but the bars are too small to label.

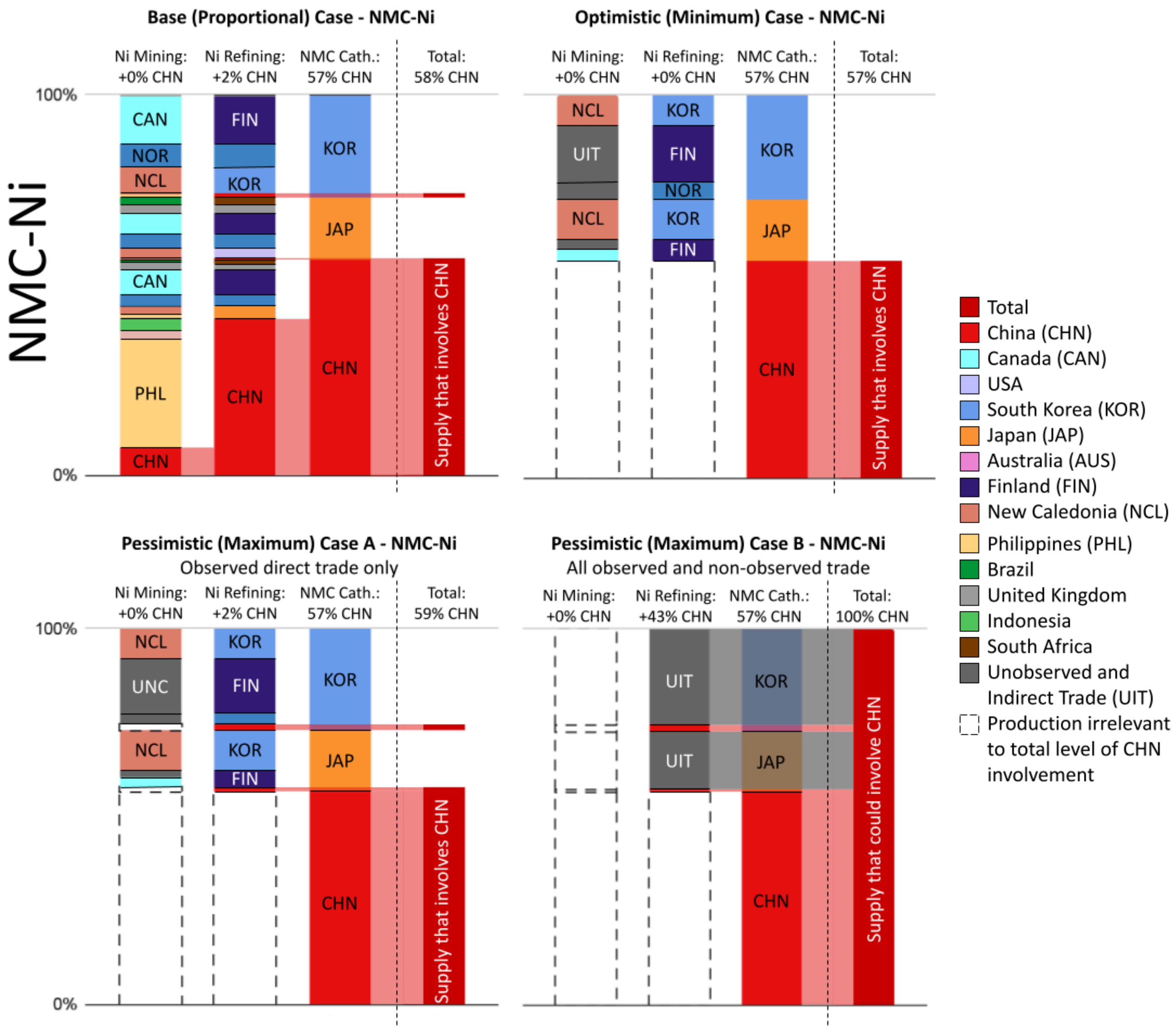

Supplementary Fig. 5. Visualization of a vulnerability index for the global NMC (Lithium Nickel Manganese Cobalt) cathode supply, for a nickel supply chain disruption in China.

Note that Brazil, the United Kingdom, Indonesia, and South Africa are represented on these diagrams, as identified in the color legend on the right hand side, but the bars are too small to label.

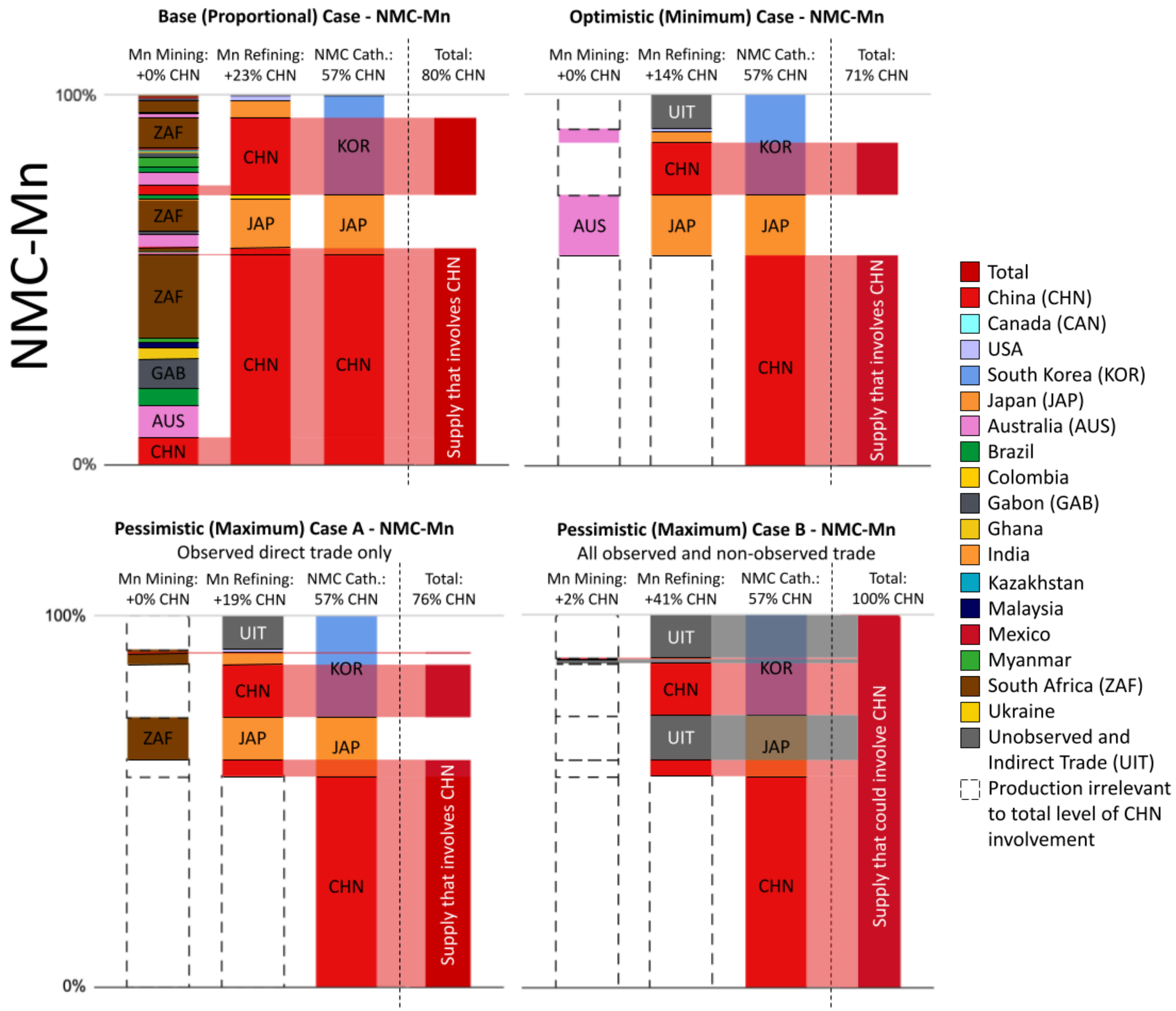

Supplementary Fig. 6. Visualization of a vulnerability index for the global NMC (Lithium Nickel Manganese Cobalt) cathode supply, for a manganese supply chain disruption in China. A constraint placed on the uncertain flows at the mining step results in less than 100% dependence, even though the uncertainty would otherwise allow the vulnerability index to approach 100%. This is because the maximum amount of uncertain trade assigned to the country of focus at each step is limited to the maximum amount of trade that the country of focus exports to countries that are not known to be involved in the next step of the supply chain. Note that the United States, Brazil, Colombia, Ghana, India, Kazakhstan, Malaysia, Mexico, Myanmar, and Ukraine are represented on these diagrams, as identified in the color legend on the right hand side, but the bars are too small to label.

Supplementary Text S4-2. Further results for multiple countries and potential trade blocs

In Supplementary Fig. 7. and Supplementary Fig. 8., we present additional illustrative results, similar to Fig. 5, that describe minimum, proportional, and maximum vulnerability to other sets of countries and regions.

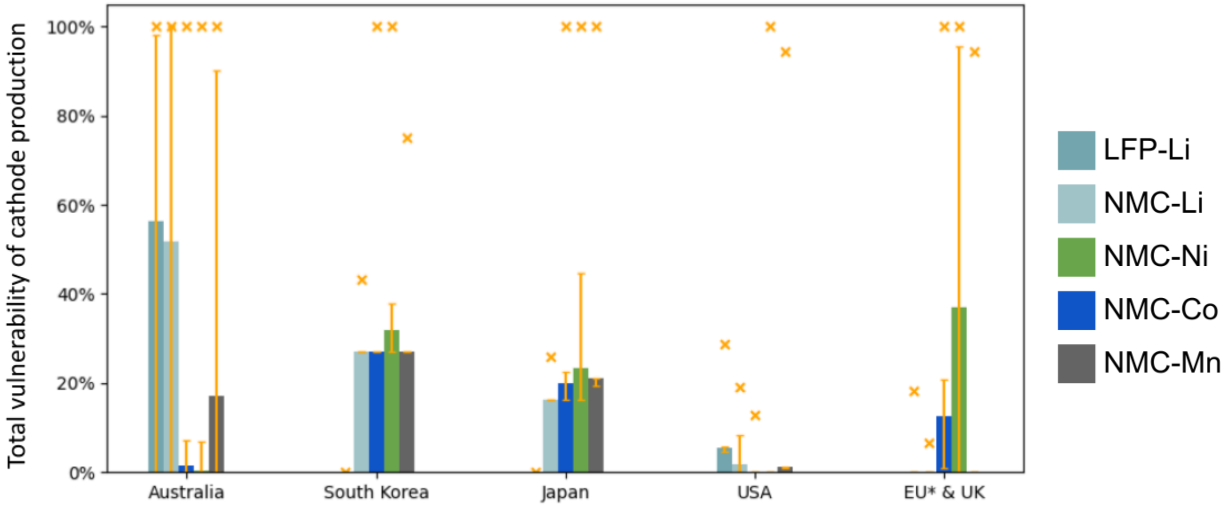

Supplementary Fig. 7. Calculated vulnerability indices for a set of "western-aligned" countries and regions.

Note EU* represents countries in the European Union and European Free Trade Association. Bars show results using proportionality assumptions, error bars (in yellow) show range from optimistic (minimum) and pessimistic (maximum) based on uncertainty about intracountry flows, and the yellow ✕ symbols show extreme upper bounds assuming all missing data originate from the focal country.

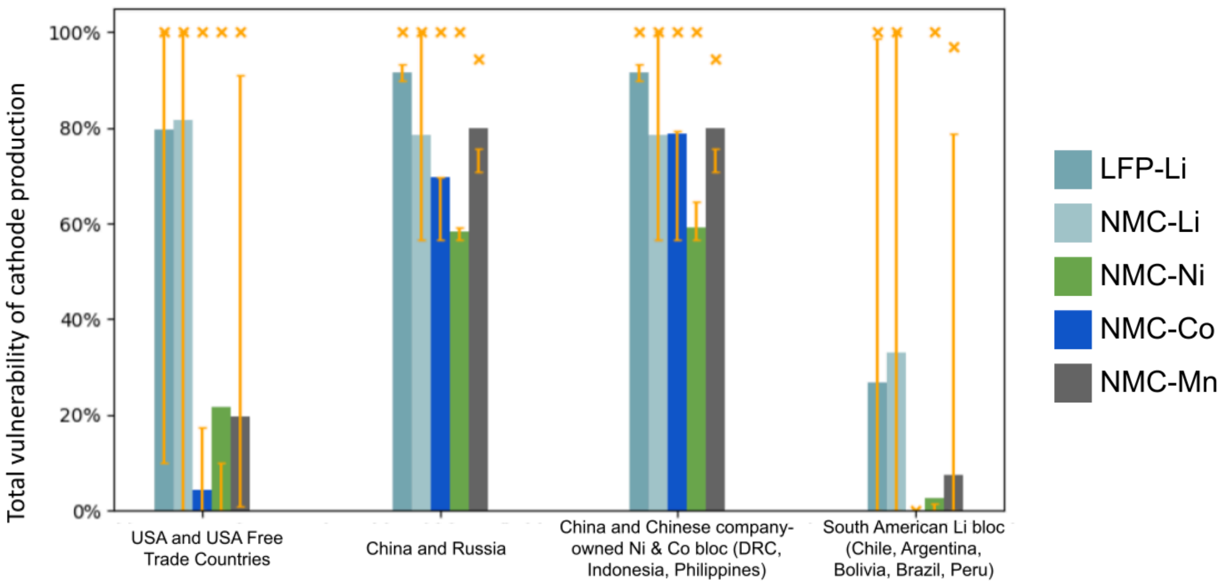

Supplementary Fig. 8. Calculated vulnerability indices for a set of potential future trade blocs. Bars show results using proportionality assumptions, error bars (in yellow) show range from optimistic (minimum) and pessimistic (maximum) based on uncertainty about intracountry flows, and the yellow ✕ symbols show extreme upper bounds assuming all missing data originate from the focal country.

References for Supplementary Information

1. Sun, X., Hao, H., Zhao, F. & Liu, Z. Tracing global lithium flow: A trade-linked material flow analysis. *Resour. Conserv. Recycl.* **124**, 50–61 (2017).
2. Sun, X., Hao, H., Liu, Z., Zhao, F. & Song, J. Tracing global cobalt flow: 1995–2015. *Resour. Conserv. Recycl.* **149**, 45–55 (2019).
3. Baars, J., Domenech, T., Bleischwitz, R., Melin, H. E. & Heidrich, O. Circular economy strategies for electric vehicle batteries reduce reliance on raw materials. *Nat. Sustain.* **4**, 71–79 (2021).
4. Sun, X., Hao, H., Zhao, F. & Liu, Z. Global Lithium Flow 1994–2015: Implications for Improving Resource Efficiency and Security. *Environ. Sci. Technol.* **52**, 2827–2834 (2018).
5. Hache, E., Seck, G. S., Simoen, M., Bonnet, C. & Carcanague, S. Critical raw materials and transportation sector electrification: A detailed bottom-up analysis in world transport. *Appl. Energy* **240**, 6–25 (2019).
6. International Energy Agency. *The Role of Critical Minerals in Clean Energy Transitions*. 287 https://www.iea.org/reports/the-role-of-critical-minerals-in-clean-energy-transitions (2021).
7. Harrison, D. & Ludwig, C. Electric Vehicle Battery Supply Chain Analysis. https://www.automotivelogistics.media/download?ac=291830 (2021).
8. Zhou, Y., Gohlke, D., Rush, L., Kelly, J. & Dai, Q. *Lithium-Ion Battery Supply Chain for E-Drive Vehicles in the United States: 2010–2020*. https://www.osti.gov/biblio/1778934 (2021) doi:10.2172/1778934.
9. Kane, M. Ford Secured Battery Contracts For Mass Electrification, Adds LFP Chemistry. *InsideEVs* https://insideevs.com/news/599791/ford-secured-battery-contracts-lfp-chemistry/ (2022).
10. Unglesbee, B. GM investing $650M to develop Nevada lithium mine. *Supply Chain Dive* https://www.supplychaindive.com/news/gm-investing-650m-lithium-Thacker-Pass-mine-Nev

ada-Lithium-Americas/641599/ (2023).

11. Lambert, F. Tesla releases list of battery material suppliers, confirms long-term nickel deal with Vale. *Electrek* https://electrek.co/2022/05/06/tesla-list-battery-material-suppliers-long-term-nickel-deal-vale/ (2022).
12. Helbig, C., Bradshaw, A. M., Wietschel, L., Thorenz, A. & Tuma, A. Supply risks associated with lithium-ion battery materials. *J. Clean. Prod.* **172**, 274–286 (2018).
13. Wentker, M., Greenwood, M. & Leker, J. A Bottom-Up Approach to Lithium-Ion Battery Cost Modeling with a Focus on Cathode Active Materials. *Energies* **12**, 504 (2019).
14. Olivetti, E. A., Ceder, G., Gaustad, G. G. & Fu, X. Lithium-Ion Battery Supply Chain Considerations: Analysis of Potential Bottlenecks in Critical Metals. *Joule* **1**, 229–243 (2017).
15. Spears, B. M., Brownlie, W. J., Cordell, D., Hermann, L. & Mogollón, J. M. Concerns about global phosphorus demand for lithium-iron-phosphate batteries in the light electric vehicle sector. *Commun. Mater.* **3**, 1–2 (2022).
16. Hotter, A. Don't forget phosphate when securing critical raw materials for electrification | Hotter Commodities. *Fastmarkets* https://www.fastmarkets.com/insights/dont-forget-phosphate-for-electrification-andrea-hotter (2023).
17. USGS. *Mineral Commodity Summaries 2022*. https://doi.org/10.3133/mcs2022 (2022).
18. Xu, C. *et al.* Reply to: Concerns about global phosphorus demand for lithium-iron-phosphate batteries in the light electric vehicle sector. *Commun. Mater.* **3**, 1–2 (2022).
19. Valero, A., Valero, A., Calvo, G. & Ortego, A. Material bottlenecks in the future development of green technologies. *Renew. Sustain. Energy Rev.* **93**, 178–200 (2018).
20. Greenwood, M., Wentker, M. & Leker, J. A region-specific raw material and lithium-ion battery criticality methodology with an assessment of NMC cathode technology. *Appl.*

Energy **302**, 117512 (2021).

21. Ballinger, B. *et al.* The vulnerability of electric vehicle deployment to critical mineral supply. *Appl. Energy* **255**, 113844 (2019).
22. Sun, X., Liu, Z., Zhao, F. & Hao, H. Global Competition in the Lithium-Ion Battery Supply Chain: A Novel Perspective for Criticality Analysis. *Environ. Sci. Technol.* **55**, 12180–12190 (2021).
23. Scott, S. & Ireland, R. *Lithium-Ion Battery Materials for Electric Vehicles and Their Global Value Chains*. 42 (2020).
24. Matos, C. *et al. Material System Analysis of Five Battery Related Raw Materials: Cobalt, Lithium, Manganese, Natural Graphite, Nickel*. https://ntnuopen.ntnu.no/ntnu-xmlui/bitstream/handle/11250/2686018/Matos%2bet%2bal%2bb2020%2b-%2bMSA%2bof%2bfive%2bbattery-related%2braw%2bmaterials.pdf (2020).
25. Sun, X., Hao, H., Hartmann, P., Liu, Z. & Zhao, F. Supply risks of lithium-ion battery materials: An entire supply chain estimation. *Mater. Today Energy* **14**, 100347 (2019).
26. Granholm, J. M. *National Blueprint for Lithium Batteries 2021-2030*. 24 https://www.energy.gov/sites/default/files/2021-06/FCAB%20National%20Blueprint%20Lithium%20Batteries%200621_0.pdf (2021).
27. Hund, K., Porta, D. L., Fabregas, T. P., Laing, T. & Drexhage, J. *The Mineral Intensity of the Clean Energy Transition*. 112 (2020).
28. Bauer, D. J., Nguyen, R. T. & Smith, B. J. *Critical Materials Assessment*. https://www.energy.gov/sites/default/files/2023-07/doe-critical-material-assessment_07312023.pdf (2023).
29. 2022 Final List of Critical Minerals. *Federal Register* https://www.federalregister.gov/documents/2022/02/24/2022-04027/2022-final-list-of-critical-minerals (2022).
30. International Fertilizer Association (IFA). Phosphate Products. IFASTAT (2023).

31. Sovacool, B. K. *et al.* Sustainable minerals and metals for a low-carbon future. *Science* **367**, 30–33 (2020).

32. Houache, M. S. E., Yim, C.-H., Karkar, Z. & Abu-Lebdeh, Y. On the Current and Future Outlook of Battery Chemistries for Electric Vehicles—Mini Review. *Batteries* **8**, 70 (2022).

33. Miao, Y., Hynan, P., von Jouanne, A. & Yokochi, A. Current Li-Ion Battery Technologies in Electric Vehicles and Opportunities for Advancements. *Energies* **12**, 1074 (2019).

34. Nitta, N., Wu, F., Lee, J. T. & Yushin, G. Li-ion battery materials: present and future. *Mater. Today* **18**, 252–264 (2015).

35. Raker, B. Sodium-ion batteries are gaining traction thanks to a 'game-changing' new partnership: 'The battery of the future'. *Yahoo News* https://news.yahoo.com/sodium-ion-batteries-gaining-traction-150000318.html (2023).

36. Rudisuela, K. Battle of the batteries - cost versus performance. https://nickelinstitute.org/en/blog/2020/june/battle-of-the-batteries-cost-versus-performance/ (2020).

37. Hanley, S. LFP Battery News -- Is The End Of Nickel In Sight? *CleanTechnica* https://cleantechnica.com/2022/03/31/lfp-battery-news-is-the-end-of-nickel-in-sight/ (2022).

38. Knehr, K., Kubal, J., Nelson, P. & Ahmed, S. *Battery Performance and Cost Modeling for Electric-Drive Vehicles: A Manual for BatPaC v5.0*. ANL/CSE-22/1, 1877590, 176234 https://www.osti.gov/servlets/purl/1877590/ (2022) doi:10.2172/1877590.

39. Downs, E. S. The Chinese Energy Security Debate. *China Q.* **177**, 21–41 (2004).

40. Kitamura, T. & Managi, S. Energy security and potential supply disruption: A case study in Japan. *Energy Policy* **110**, 90–104 (2017).

41. Paust, J. J. & Blaustein, A. P. The Arab Oil Weapon—A Threat to International Peace. *Am. J. Int. Law* **68**, 410–439 (1974).

42. Vivoda, V. Evaluating energy security in the Asia-Pacific region: A novel methodological approach. *Energy Policy* **38**, 5258–5263 (2010).

43. Yan, W. *et al.* Rethinking Chinese supply resilience of critical metals in lithium-ion batteries. *J. Clean. Prod.* **256**, 120719 (2020).
44. Lee, J. *et al.* Reviewing the material and metal security of low-carbon energy transitions. *Renew. Sustain. Energy Rev.* **124**, 109789 (2020).
45. Zhang, J., Liang, C. & Dunn, J. B. Graphite Flows in the U.S.: Insights into a Key Ingredient of Energy Transition. *Environ. Sci. Technol.* **57**, 3402–3414 (2023).
46. van den Brink, S., Kleijn, R., Sprecher, B. & Tukker, A. Identifying supply risks by mapping the cobalt supply chain. *Resour. Conserv. Recycl.* **156**, 104743 (2020).
47. Nakajima, K. *et al.* Global distribution of material consumption_ Nickel, copper, and iron | Elsevier Enhanced Reader. *Resour. Conserv. Recycl.* **133**, 369–374 (2018).
48. Sanfélix, J., De la Rúa, C., Schmidt, J. H., Messagie, M. & Van Mierlo, J. Environmental and economic performance of an li-ion battery pack: A multiregional input-output approach. *Energies* **9**, 584 (2016).
49. Hawkins, T., Hendrickson, C., Higgins, C., Matthews, H. S. & Suh, S. A mixed-unit input-output model for environmental life-cycle assessment and material flow analysis. *Environ. Sci. Technol.* **41**, 1024–1031 (2007).
50. Cobas-Flores, E., Hendrickson, C., Lave, L. B. & McMichael, F. C. Life cycle analysis of batteries using economic input-output analysis. in *Proceedings of the 1996 IEEE International Symposium on Electronics and the Environment. ISEE-1996* 130–134 (IEEE, 1996).
51. Hao, H. *et al.* Multi-layer networks research on analyzing supply risk transmission of lithium industry chain. *Resour. Policy* **79**, 102933 (2022).
52. Wang, X., Wang, A. & Zhu, D. Simulation Analysis of Supply Crisis Propagation Based on Global Nickel Industry Chain. *Front. Energy Res.* **10**, (2022).
53. Wang, X., Li, H., Yao, H., Zhu, D. & Liu, N. Simulation analysis of the spread of a supply crisis based on the global natural graphite trade network. *Resour. Policy* **59**, 200–209

(2018).

54. Sun, X., Shi, Q. & Hao, X. Supply crisis propagation in the global cobalt trade network. *Resour. Conserv. Recycl.* **179**, 106035 (2022).
55. *The Routledge Handbook of Energy Security*. (Routledge, 2010). doi:10.4324/9780203834602.
56. Willrich, M. International Energy Issues and Options. *Annu. Rev. Energy* **1**, 743–772 (1976).
57. Cherp, A. & Jewell, J. The concept of energy security: Beyond the four As. *Energy Policy* **75**, 415–421 (2014).
58. Vakulchuk, R., Overland, I. & Scholten, D. Renewable energy and geopolitics: A review. *Renew. Sustain. Energy Rev.* **122**, 109547 (2020).
59. Su, C.-W., Khan, K., Umar, M. & Zhang, W. Does renewable energy redefine geopolitical risks? *Energy Policy* **158**, 112566 (2021).
60. Schrijvers, D. *et al.* A review of methods and data to determine raw material criticality. *Resour. Conserv. Recycl.* **155**, 104617 (2020).
61. Erdmann, L. & Graedel, T. E. Criticality of Non-Fuel Minerals: A Review of Major Approaches and Analyses. *Environ. Sci. Technol.* **45**, 7620–7630 (2011).
62. Helbig, C., Bruckler, M., Thorenz, A. & Tuma, A. An overview of indicator choice and normalization in raw material supply risk assessments. *Resources* **10**, (2021).
63. Graedel, T. E., Harper, E. M., Nassar, N. T., Nuss, P. & Reck, B. K. Criticality of metals and metalloids. *Proc. Natl. Acad. Sci.* **112**, 4257–4262 (2015).
64. Frenzel, M., Kullik, J., Reuter, M. A. & Gutzmer, J. Raw material ‘criticality’—sense or nonsense? *J. Phys. Appl. Phys.* **50**, 123002 (2017).
65. Sun, X., Hao, H., Liu, Z. & Zhao, F. Insights into the global flow pattern of manganese. *Resour. Policy* **65**, 101578 (2020).
66. Trost, J. N. & Dunn, J. B. Assessing the feasibility of the Inflation Reduction Act’s EV critical mineral targets. *Nat. Sustain.* 1–5 (2023) doi:10.1038/s41893-023-01079-8.

67. Leontief, W. *Input-Output Economics*. (Oxford University Press, 1986).

68. Ayres, R. U. & Kneese, A. V. Production, Consumption, and Externalities. *Am. Econ. Rev.* **59**, 282–297 (1969).

69. Nakajima, K. *et al.* Global supply chain analysis of nickel: importance and possibility of controlling the resource logistics. *Metall. Res. Technol.* **111**, 339–346 (2014).

70. Igogo, T. A., Sandor, D. L., Mayyas, A. T. & Engel-Cox, J. *Supply Chain of Raw Materials Used in the Manufacturing of Light-Duty Vehicle Lithium-Ion Batteries*. NREL/TP-6A20-73374, 1560124 http://www.osti.gov/servlets/purl/1560124/ (2019) doi:10.2172/1560124.

71. Kravchenko, A. *Where and How to Dodge Taxes and Shift Money Abroad Using Trade Misinvoicing : A Beginner's Guide*. https://repository.unescap.org/bitstream/handle/20.500.12870/1176/ESCAP-2018-WP-Where-and-how-to-dodge-taxes-and-shift-money-abroad-using-trade-misinvoicing.pdf?sequence=1&isAllowed=y (2018).

72. Konijn, P., de Boer, S. & van Dalen, J. Input-output analysis of material flows with application to iron, steel and zinc. *Struct. Change Econ. Dyn.* **8**, 129–153 (1997).

73. Daniel, S. E., Pappis, C. P. & Voutsinas, T. G. Applying life cycle inventory to reverse supply chains: a case study of lead recovery from batteries. *Resour. Conserv. Recycl.* **37**, 251–281 (2003).

74. Lee, K.-M. *et al.* Impact of the Topology of Global Macroeconomic Network on the Spreading of Economic Crises. *PLOS ONE* **6**, e18443 (2011).

75. Schrijver, A. On the history of the transportation and maximum flow problems. *Math. Program.* **91**, 437–445 (2002).

76. Edmonds, J. & Karp, R. M. Theoretical Improvements in Algorithmic Efficiency for Network Flow Problems. *J. Assoc. Comput. Mach.* **19**, 248–264 (1972).

77. LaRocca, G. M. Global Value Chains: Lithium in Lithium-ion Batteries for Electric Vehicles.

39 (2020).

78. Shi, Q., Sun, X., Xu, M. & Wang, M. The multiplex network structure of global cobalt industry chain. *Resour. Policy* **76**, 102555 (2022).
79. Matthews, D. Global Value Chains: Cobalt in Lithium-ion Batteries for Electric Vehicles. https://www.usitc.gov/publications/332/working_papers/id_wp_cobalt_final_052120-compliant.pdf (2020).
80. IntraCen. Trade Map - Trade statistics for international business development. *TradeMap* https://www.trademap.org/Index.aspx (2022).
81. Markhonko, V. Asymmetries in official international trade statistics and analysis of globalization. *Int. Conf. Meas. Int. Trade Econ. Glob.* (2014).
82. Ronzheimer, I., Lima, J. D., Budnevich, C. & Gomies, M. *Towards the Measurement of Electromobility in International Trade*. 118 (2022).
83. Tian, X. *et al.* Features of critical resource trade networks of lithium-ion batteries. *Resour. Policy* **73**, 102177 (2021).
84. Cobalt Institute. Cobalt Use. *Cobalt Institute* https://www.cobaltinstitute.org/about-cobalt/cobalt-life-cycle/cobalt-use/ (2022).
85. Nickel Institute. About nickel. *Nickel Institute* https://nickelinstitute.org/en/about-nickel-and-its-applications/#04-first-use-nickel (2022).
86. Chen, Y. & Chen, M. Evolution of the global phosphorus trade network: A production perspective on resilience. *J. Clean. Prod.* **405**, 136843 (2023).
87. Li, B. *et al.* Network evolution and risk assessment of the global phosphorus trade. *Sci. Total Environ.* **860**, 160433 (2023).
88. Xu, C. *et al.* Future material demand for automotive lithium-based batteries. *Commun. Mater.* **1**, 1–10 (2020).
89. Denina, C. Miners face supply chain overhaul to meet U.S. EV credit deadline. *Reuters* (2022).

90. International Energy Agency. *Global Supply Chains of EV Batteries*. (2022).

91. Burrell, R. China’s stronghold grip on cathodes set to near 90% by 2030 | Benchmark Source. *Benchmark Mineral Intelligence* https://source.benchmarkminerals.com/article/chinas-stronghold-grip-on-cathodes-set-to-near-90-by-2030 (2022).
