# Supplementary material for: Electric vehicle battery chemistry affects supply chain disruption vulnerabilities
Source: Nat Commun. 2024 Mar 8;15:2143. doi: 10.1038/s41467-024-46418-1 (PMC10923860; doi:10.1038/s41467-024-46418-1)
Supplement: Supplementary file 3 — Reporting Summary [file 41467_2024_46418_MOESM3_ESM.pdf]

nature portfolio

Corresponding author(s): Jeremy Michalek

Last updated by author(s): 2023 10 05

Reporting Summary

Nature Portfolio wishes to improve the reproducibility of the work that we publish. This form provides structure for consistency and transparency in reporting. For further information on Nature Portfolio policies, see our Editorial Policies and the Editorial Policy Checklist.

Statistics

For all statistical analyses, confirm that the following items are present in the figure legend, table legend, main text, or Methods section.

n/a	Confirmed	
☒	☐	The exact sample size (*n*) for each experimental group/condition, given as a discrete number and unit of measurement
☒	☐	A statement on whether measurements were taken from distinct samples or whether the same sample was measured repeatedly
☒	☐	The statistical test(s) used AND whether they are one- or two-sided<br>*Only common tests should be described solely by name; describe more complex techniques in the Methods section.*
☒	☐	A description of all covariates tested
☒	☐	A description of any assumptions or corrections, such as tests of normality and adjustment for multiple comparisons
☒	☐	A full description of the statistical parameters including central tendency (e.g. means) or other basic estimates (e.g. regression coefficient) AND variation (e.g. standard deviation) or associated estimates of uncertainty (e.g. confidence intervals)
☒	☐	For null hypothesis testing, the test statistic (e.g. *F*, *t*, *r*) with confidence intervals, effect sizes, degrees of freedom and *P* value noted<br>*Give P values as exact values whenever suitable.*
☒	☐	For Bayesian analysis, information on the choice of priors and Markov chain Monte Carlo settings
☒	☐	For hierarchical and complex designs, identification of the appropriate level for tests and full reporting of outcomes
☒	☐	Estimates of effect sizes (e.g. Cohen's *d*, Pearson's *r*), indicating how they were calculated

Our web collection on statistics for biologists contains articles on many of the points above.

Software and code

Policy information about availability of computer code

Data collection	No special software was used to collect the data; data was directly downloaded or accessed from the sources cited (IntraCen's TradeMap, the USGS Mineral Commodity Survey, Sun et al. 2021's supplementary materials, and IFAStat regional production data.
Data analysis	Custom code ("SankeyData.py") was created to analyze the aforementioned data to generate a material flow analysis in the form of a Sankey diagram and associated material flows comma separated values file. This material flow data was then analyzed with further custom code (VulnerabilityCalc.py) to measure the vulnerability index described in the paper.

For manuscripts utilizing custom algorithms or software that are central to the research but not yet described in published literature, software must be made available to editors and reviewers. We strongly encourage code deposition in a community repository (e.g. GitHub). See the Nature Portfolio guidelines for submitting code & software for further information.

Data

Policy information about availability of data

All manuscripts must include a data availability statement. This statement should provide the following information, where applicable:

- Accession codes, unique identifiers, or web links for publicly available datasets
- A description of any restrictions on data availability
- For clinical datasets or third party data, please ensure that the statement adheres to our policy

Source data are provided with this paper; we only use publicly available production and trade data for this analysis, from the U.S. Geological Service’s Mineral

Commodity Survey, Sun et al. 2021, International Fertilizer Association, and IntraCen's TradeMap, which is described in detail in Appendix C2. Data Sources. All data files used in this analysis, including both raw data and processed data, are freely available in the Github repository linked to this paper (https://github.com/acheng98/ev-battery-chemistry-supply-chain-vulnerabilities).

Research involving human participants, their data, or biological material

Policy information about studies with human participants or human data. See also policy information about sex, gender (identity/presentation), and sexual orientation and race, ethnicity and racism.

Reporting on sex and gender	N/A
Reporting on race, ethnicity, or other socially relevant groupings	N/A
Population characteristics	N/A
Recruitment	N/A
Ethics oversight	N/A

Note that full information on the approval of the study protocol must also be provided in the manuscript.

Field-specific reporting

Please select the one below that is the best fit for your research. If you are not sure, read the appropriate sections before making your selection.

☐ Life sciences ☐ Behavioural & social sciences ☒ Ecological, evolutionary & environmental sciences

For a reference copy of the document with all sections, see nature.com/documents/nr-reporting-summary-flat.pdf

Ecological, evolutionary & environmental sciences study design

All studies must disclose on these points even when the disclosure is negative.

Study description	This study observed 2020 battery material supply chains for lithium, nickel, cobalt, manganese, and phosphorus, for use in LFP and NMC battery cathode production. Sankey diagrams were created representing a material flow analysis, which were then analyzed with a custom 'vulnerability index' method that involved standard network analysis algorithms (depth first search, breadth first search, maximum flow optimization).
Research sample	We only use publicly available production and trade data for this analysis, from the U.S. Geological Service's Mineral Commodity Survey, Sun et al. 2021, International Fertilizer Association, and IntraCen's TradeMap, which is described in detail in Appendix C2. Data Sources. All data files used in this analysis, including both raw data and processed data, are freely available in the Github repository linked to this paper (https://github.com/acheng98/ev-battery-chemistry-supply-chain-vulnerabilities).
Sampling strategy	No sample size calculation was used as this is an observational study of 2020 battery material supply chains.
Data collection	No special software was used to collect the data; data was directly downloaded or accessed from the sources cited (IntraCen's TradeMap, the USGS Mineral Commodity Survey, Sun et al. 2021's supplementary materials, and IFAStat regional production data.
Timing and spatial scale	The data collected for this analysis was primarily collected in June 2022 for Lithium, Nickel, Cobalt, and Manganese trade codes and production data from the aforementioned sources and reverified in October 2023. The phosphorus trade and production data was collected October 2023.
Data exclusions	No data in the data gathered was excluded from the analysis.
Reproducibility	Given the nature of the code and data source, the study and results are easily reproducible given installation of the python programming language.
Randomization	Randomization is not relevant to the study as it is an observational study of 2020 battery material supply chains.
Blinding	Blinding is not relevant to the study as it is an observational study of 2020 battery material supply chains.

Did the study involve field work? ☐ Yes ☒ No

Reporting for specific materials, systems and methods

We require information from authors about some types of materials, experimental systems and methods used in many studies. Here, indicate whether each material, system or method listed is relevant to your study. If you are not sure if a list item applies to your research, read the appropriate section before selecting a response.

Materials & experimental systems

n/a	Involved in the study
☒	☐ Antibodies
☒	☐ Eukaryotic cell lines
☒	☐ Palaeontology and archaeology
☒	☐ Animals and other organisms
☒	☐ Clinical data
☒	☐ Dual use research of concern
☒	☐ Plants

Methods

n/a	Involved in the study
☒	☐ ChIP-seq
☒	☐ Flow cytometry
☒	☐ MRI-based neuroimaging
